# Phalangeal cortical bone distribution reveals different dexterous and climbing behaviors in *Australopithecus sediba* and *Homo naledi*

**DOI:** 10.1126/sciadv.adt1201

**Published:** 2025-05-14

**Authors:** Samar M. Syeda, Christopher J. Dunmore, Matthew M. Skinner, Lee R. Berger, Steven E. Churchill, Bernhard Zipfel, Tracy L. Kivell

**Affiliations:** ^1^ Richard Gilder Graduate School, American Museum of Natural History, New York, NY 10024, USA.; ^2^ Division of Anthropology, American Museum of Natural History, New York, NY 10024, USA.; ^3^ Department of Human Origins, Max Planck Institute for Evolutionary Anthropology, Leipzig, Germany.; ^4^ School of Biosciences, University of Kent, Canterbury, U.K.; ^5^ Centre for the Exploration of the Deep Human Journey, School of Anatomical Sciences, University of the Witwatersrand, Private Bag 3, Wits 2050, South Africa.; ^6^ The National Geographic Society, 1145 17th St NW, Washington DC 20036, USA.; ^7^ The Carnegie Institution for Science, 5251 Broad Branch Rd NW, Washington, DC 20015, USA.; ^8^ Department of Evolutionary Anthropology, Duke University, Durham, NC 27710, USA.; ^9^ Evolutionary Studies Institute, University of the Witwatersrand, Johannesburg, South Africa.

## Abstract

The evolution of the human hand is marked by a transition from a hand primarily used for locomotion to one primarily used for dexterous manipulation. The hand skeletons of Plio-Pleistocene hominins have different mosaics of human-like features associated with enhanced dexterity and ape-like features associated with locomotor hand use. However, the functional relevance of the ape-like features is debated, particularly due to a lack of complete and associated hand remains. Here, we investigate the internal phalangeal cortical structure of the nearly complete *Australopithecus sediba* MH2 hand and *Homo naledi* hand 1 to provide both insight into the manual behaviors of these fossil hominins and functional clarity regarding the mosaic features found within their hands. The phalangeal cortical structure demonstrates diversity in Plio-Pleistocene hand use, with *A. sediba* and *H. naledi* each indicating different dexterous abilities and different climbing strategies, supporting the functional importance of the ape-like features.

## Teaser

Phalangeal bone structure provides evidence for variable locomotor strategies across South African fossil hominins.

## INTRODUCTION

The hand morphology of Plio-Pleistocene hominins is generally thought to record a functional shift from a hand used for primarily locomotion to a hand used primarily for manipulation [e.g., (*1*, *2*)]. When, how, and how many times this functional shift occurred is challenging to reconstruct as the morphological features typically associated with modern human-like dexterity are often present alongside ape-like features typically associated with arboreal locomotion, such as climbing and suspension (*3*–*12*). In addition, the fossil record of relatively complete hand skeletons is poor. Comparative studies of available specimens have revealed a notable degree of external and internal morphological variation in hand remains of *Australopithecus*, *Paranthropus*, and *Homo* that suggests diversity in manual behaviors throughout the Plio-Pleistocene [e.g., (*6*, *13*–*18*)]. Within this morphological diversity, the importance of ape-like climbing features is debated, as it is unclear whether these traits are retained from an arboreal ancestor or functional morphology. This affects our understanding of how human dexterity evolved, particularly whether it developed alongside hand use for locomotion or only after it ceased.

This morphological variation is especially prominent in the South African hominins, partly because South African sites also preserve some of the most complete hand skeletons. While these fossils come from a restricted geographical area in the Cradle of Humankind, they span a large temporal range: *Australopithecus* “*prometheus*” StW 573 [~3.67 to 2.2 million years (Ma)] (*19*, *20*) and *Australopithecus africanus* from Sterkfontein [~3.3 to 2.1 Ma] (*19*–*21*), *Australopithecus sediba* from Malapa (1.98 Ma) (*22*), isolated fossils potentially attributed to *Paranthropus robustus* or early *Homo* at Swartkrans (~2.3 to 1.0 Ma) (*23*–*25*), and *Homo naledi* within the Rising Star cave system (~241 to 335 ka) (*26*, *27*). Apart from the StW 573 hand skeleton that has yet to be formally described (*28*), each remaining taxon demonstrates a mix of external morphological features that is distinct not only from extant hominids but also from other fossil hominins. This mosaic of functional signals is also true for studies of the internal cortical and trabecular bone structure within the wrist and metacarpal bones (*13*, *15*, *17*), which, together with external morphological variation, raises questions about the specific locomotor and/or manipulative behaviors of these South African hominins.

Here, we provide further functional resolution to our understanding of the evolution of hominin hand use by investigating the internal bone structure of the proximal and intermediate phalanges of South African hominins, with a focus on the almost complete, associated hand skeletons of *A. sediba* and *H. naledi*. We focus on the phalanges because (i) they are the first point of contact with the substrate during locomotion or the object during manipulation and are consistently loaded during these behaviors; (ii) their morphology is linked with the degree of arboreality in extant primates (*29*–*31*) and thus key in behavioral interpretations of arboreality in fossil hominin taxa [e.g., (*5*, *32*–*34*) but see (*35*)].

Across the South African fossil hominins, hand remains attributed to *A. africanus* and to either *P. robustus* or early *Homo* include several isolated specimens that cannot be associated with each other or other fossil remains (*7*, *10*, *36*, *37*). In contrast, the hand remains of *A. sediba* Malapa Hominin (MH) 2 and *H. naledi* hand 1 were found in semi-articulation, are associated and each from a single individual (*5*, *6*). The *A. sediba* right hand is further associated with a complete right upper limb and partial skeleton of MH2 (*6*, *38*), while *H. naledi* hand 1 is associated with numerous upper limb and other postcranial remains from multiple individuals (*39*, *40*). The associated hands of *A. sediba* MH2 and *H. naledi* hand 1 demonstrate different mosaics of ape-like and human-like features, suggesting hands used for both locomotion and manipulation (*5*, *6*, *15*, *34*). For example, the intrinsic hand proportions of both *A. sediba* and *H. naledi* are human-like, while the wrist and pollical metacarpals show differing functional signals in the two species (*5*, *34*). In *A. sediba*, the distribution of internal trabecular bone suggests human-like loading of the midcarpal joint and the base of the thumb, but its pollical metacarpal is gracile (*6*, *13*, *15*). In contrast, *H. naledi* has a robust pollical metacarpal and human- and Neanderthal-like radial carpometacarpal articulations, apart from an unusually small trapeziometacarpal joint (*5*, *14*).

In addition to the varying mosaics of morphologies found within the carpus and metacarpus of these two South African hominins, the manual phalanges have generally been interpreted as reflecting habitual arboreal or climbing behaviors (*5*, *6*, *13*, *15*). Although the proximal and intermediate phalanges of these species vary in shape and robusticity, *A. sediba* shows longitudinal curvature of the phalangeal shaft (both intermediate and proximal phalanges) that falls closer to the mean of African apes than to that of humans (*5*). *H. naledi* is unusual among known hominins in having both *Pan*-like curvature of the proximal phalanges and even more strongly curved intermediate phalanges that are most similar to Asian apes (*5*). Both species also show well-developed flexor sheath ridges, particularly so in the *A. sediba* intermediate phalanges (*6*, *7*). As adaptive behavioral change generally precedes evolutionary changes in morphology, it is unclear whether the “climbing” features of hominin phalanges are functionally important morphology or are neutral retentions from a more arboreal ancestor (*35*, *41*–*43*). There is persisting debate regarding the adaptive importance of primitive ape-like features and the degree of arboreality/climbing in early fossil hominins, which directly affects our interpretation of how hominin manual dexterity evolved; enhanced manual dexterity evolved either alongside locomotor hand use or was only able to evolve with the cessation of locomotor hand use. Studies of plastic “ecophenotypic” morphological traits, such as internal bone structure, can help resolve this debate. As bone plastically models and remodels throughout life in response to the magnitude and direction of load, analysis of internal bone (cortical and trabecular) structure provides more information about a bone or joint’s function and, in turn, an individual’s behavior than external morphology alone (*44*–*47*).

Our previous work has demonstrated discrete differences between cortical bone structure of humans compared to other extant hominids (the great apes) in the proximal and intermediate phalanges of the hand that reflect their manual behaviors (*48*–*50*). Therefore, within this extant hominid comparative context, we investigate the cortical bone distribution, cortical bone thickness, and bending and torsional rigidity of *A. sediba* and *H. naledi* proximal and intermediate manual phalanges, as well as in other isolated South African hominin phalangeal remains. We aim to elucidate the functional importance of climbing features and to make inferences about the habitual loading of the fingers, constituting overall hand use in each species. We assume that specimens showing a cortical bone distribution and bone rigidity (*J*) that is more similar to humans reflect habitual use of the hands primarily for manipulation, even if the dexterous abilities are more limited. In contrast, we assume that specimens that show a cortical bone distribution and bone rigidity that is more similar to extant great apes reflect frequent use of the hands for flexed-finger and thumb grasping during locomotor loading. It is important to note that locomotor and manipulation loading are not mutually exclusive; since loads incurred by human digits during stone tool use and production (*51*) are approximately six times lower than those incurred during single-arm suspension (*52*), we expect locomotor loading to override any functional signals of manipulation in hominin taxa that were still using their digits for climbing. However, a human-like signal within the phalangeal cortical bone would imply that the digits were likely not used (at least frequently) for locomotion.

## RESULTS

We assessed variation in cortical bone morphology of the phalangeal diaphysis (shafts) by quantifying (i) the distribution of cortical bone thickness (visualized as color maps), (ii) mean cortical thickness, and (iii) cross-sectional polar second moment of area (*J*; a property of bending and torsional rigidity) at 35, 50, and 65% of phalangeal length. All measures were quantified using R package Morphomap (*53*), and, when relevant (see Methods), cortical bone values were scaled by total phalangeal length to account for differences in absolute size across our sample. The scaled cortical bone thickness distribution values were also included within a principal components analysis (PCA) and canonical variates analysis (CVA) to assess variation across our sample (see Methods). Below, we present results of digits 2 to 5 combined as the results were generally similar for each digit when considered separately (figs. S2 to S5) but highlight when this was not the case.

### Cortical thickness distribution in the fingers

**Fig. 1.**
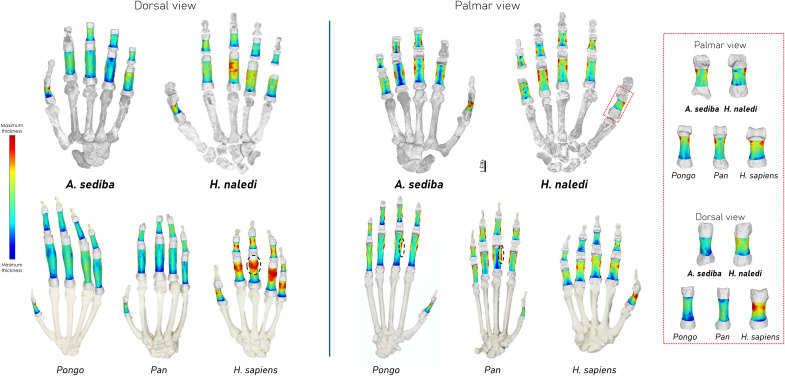
3D color maps of cortical bone distribution in fossil and extant hominin phalanges. Thickness maps are independent of each other and hand skeletons are not to scale. Thickness maps of the extant taxa represent mean models of cortical bone distribution for each taxon, and the dotted circles highlight regions of maximum cortical thickness, which in great apes is on the palmar flexor sheath ridges, while in humans, it is the dorsal shaft. Box at far right provides direct palmar and dorsal views of the PP1 cortical bone distribution.

In great ape proximal (PP) and intermediate (IP) phalanges of digits 2 to 5, cortical bone is thickest along the flexor sheath ridges of the palmar phalangeal surface, and the overall cortical bone is thicker along the palmar shaft compared to the dorsal shaft. In contrast, humans show a distinct pattern of thicker cortical bone along the dorsal shaft, rather than the palmar side (*48*–*50*). Both *A. sediba* and *H. naledi* show distinct patterns of cortical bone thickness distribution in the proximal and intermediate phalanges that are not found among our comparative sample (Fig. 1 and fig. S1).

**Fig. 2.**
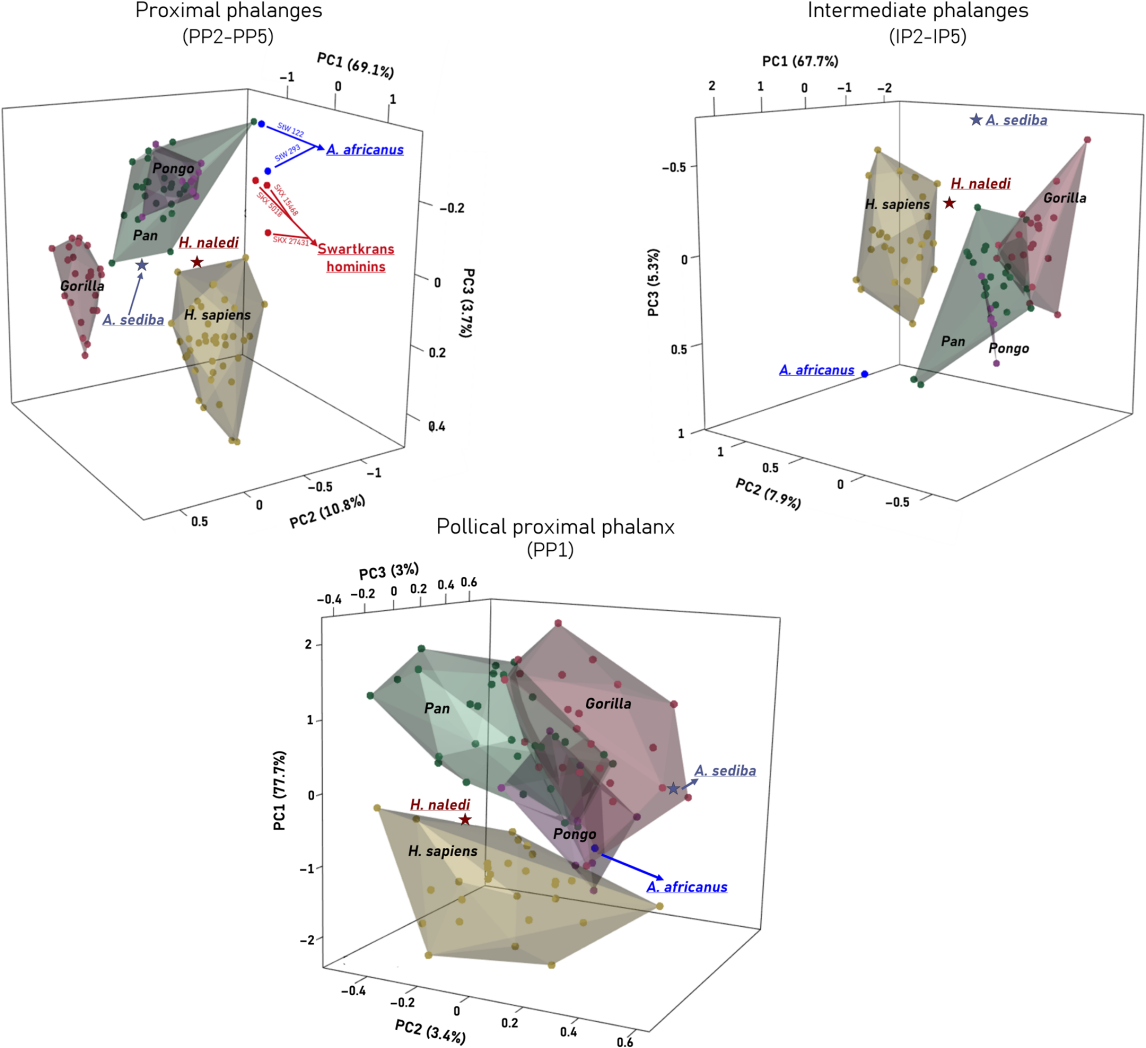
Variation in cortical bone distribution of proximal and intermediate phalanges in multivariate shape space in fossil and extant hominids. 3D PCAs depicting variation in cortical bone distribution across the proximal and intermediate phalangeal shafts. For the proximal and intermediate non-pollical phalanges, each point represents the mean cortical bone distribution pattern across all of associated phalanges (e.g., PP2 to PP5) for one individual.

In *A. sediba*, the region of thickest cortical bone in PP2-PP4 is at the peaks of the flexor sheath ridges and proximal to the trochlea. The cortical thickness of the palmar shaft, excluding the flexor ridges, is thin and intermediately thick on the dorsal shaft. PP5 is distinct with thicker cortex across the shaft and particularly at the distodorsal region. Across IPs, cortical bone is thickest across the entire length of the flexor ridges and proximal to the trochlea, with similar palmar and dorsal shaft cortical thickness in IP3-IP4 (IP2 is not well preserved), while IP5 shows slight distodorsal thickness. Together, the pattern across digits 2 to 4 is most similar to great apes, while digit 5 is distinct in showing a mixture of great ape-like and human-like cortical bone distribution. In the PCA of both the proximal and intermediate phalangeal cortical bone distribution, which capture differences in palmar and dorsal cortical thickness (PC1) as well as the proximodistal extent of the thickness (PC2), *A. sediba* falls out between the great ape distributions and the human distribution (Fig. 2 and figs. S2 and S3). The PCA of each separate digit revealed that *A. sediba* PP3 and PP4 are particularly more ape-like, falling within the *Pan* and *Gorilla* distributions, respectively (figs. S2 and S3). Results of the CVA and the, albeit low, typicality probabilities further support these results, with *A. sediba* being more similar to great apes than to humans (table S3). For both the proximal and intermediate phalanges, *A. sediba* falls far from *A. africanus* and the Swartkrans isolated phalanges and is also distinct from *H. naledi* (Fig. 1).

*H. naledi* proximal phalanges display thick cortical bone on the flexor sheath ridges, particularly so in PP3 and PP4, which is most similar to the great ape pattern. However, PP2-PP5 also have thick cortical bone along the dorsal shaft, especially in PP3, which is similar to humans. The thickness on the palmar side is localized to the midshaft-to-distal region of the bone and not the entire length of the flexor sheath ridges, and there is greater radioulnar asymmetry in the thickness patterns in PP2 and PP5. Across the IPs, cortical bone is thickest across the flexor ridges, with an intermediately thick dorsal shaft. The IP2 also displays thick cortex just proximal to the trochlea. Despite the proximal and intermediate phalanges displaying a mix of great ape- and human-like cortical bone distribution patterns, in their respective PCAs, the *H. naledi* phalanges fall out just outside the human morphospace and closer to humans than to any other fossil hominin in our sample (Fig. 2), which is also supported by the calculated typicality probabilities (table S3). This result reflects the relatively thicker dorsal cortices of the PPs and IPs compared with other fossil hominins.

### Mean cortical thickness in the fingers

**Fig. 3.**
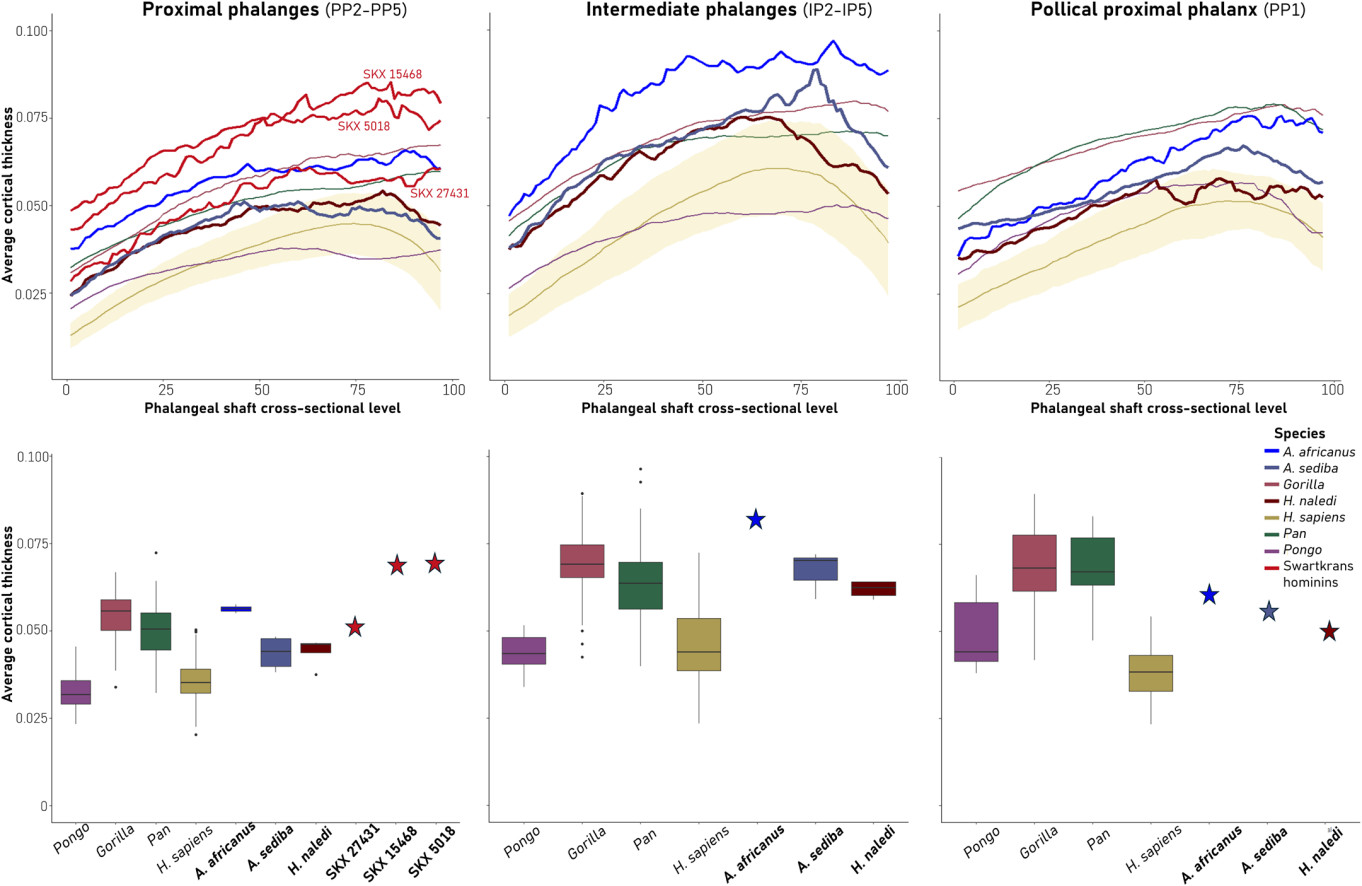
Average standardized cortical bone thickness. The top row depicts average cortical bone thickness plotted from the proximal end (0) to the distal end (100) of the defined phalangeal shaft. The shaded region around the *H. sapiens* average represents the variation in the *H. sapiens* sample. Fossil taxa are depicted with bolder lines. The bottom row depicts average cortical bone thickness of *A. sediba* and *H. naledi* proximal and intermediate phalanges in relation to our extant and fossil comparative sample. Cortical bone thickness was standardized using maximum phalangeal length.

Extant humans and great apes are distinct in the pattern of change in mean cortical thickness (standardized by bone length) across the shaft. In great apes, mean cortical thickness steadily increases proximodistally throughout the PP and IP shaft, while in humans, mean thickness also increases proximodistally, peaking at approximately 60 to 75% of shaft length, and then decreases (Fig. 3). The PPs and IPs of both *A. sediba* and *H. naledi* generally show a human-like pattern, while *A. africanus* and some of the Swartkrans phalanges show a more ape-like pattern, particularly the *A. africanus* IP. However, the *A. sediba* PP3 and PP5 show a more great ape-like pattern in which there is a general proximodistal increase in cortical thickness, which decreases only just proximal to the trochlea (figs. S4 and S5).

We also calculated standardized mean cortical thickness of the phalangeal shaft across our sample (Fig. 3). Among our extant sample and for both PPs and IPs, African apes have the highest mean cortical thickness, while humans and *Pongo* are similar with comparatively thinner cortical bone (Fig. 3). Both *A. sediba* and *H. naledi* show similar mean cortical thickness values; for the PPs, both fall out as intermediate between humans/*Pongo* and African apes, while for the IPs, both are closer to the African ape median but are in the upper range of human variation. Notably both *A. sediba* and *H. naledi* have thinner cortical bone than *A. africanus*, especially in the IPs, and the Swartkrans proximal phalanges (although SXK 27431 is more similar).

### Bending and torsional strength of the fingers

**Fig. 4.**
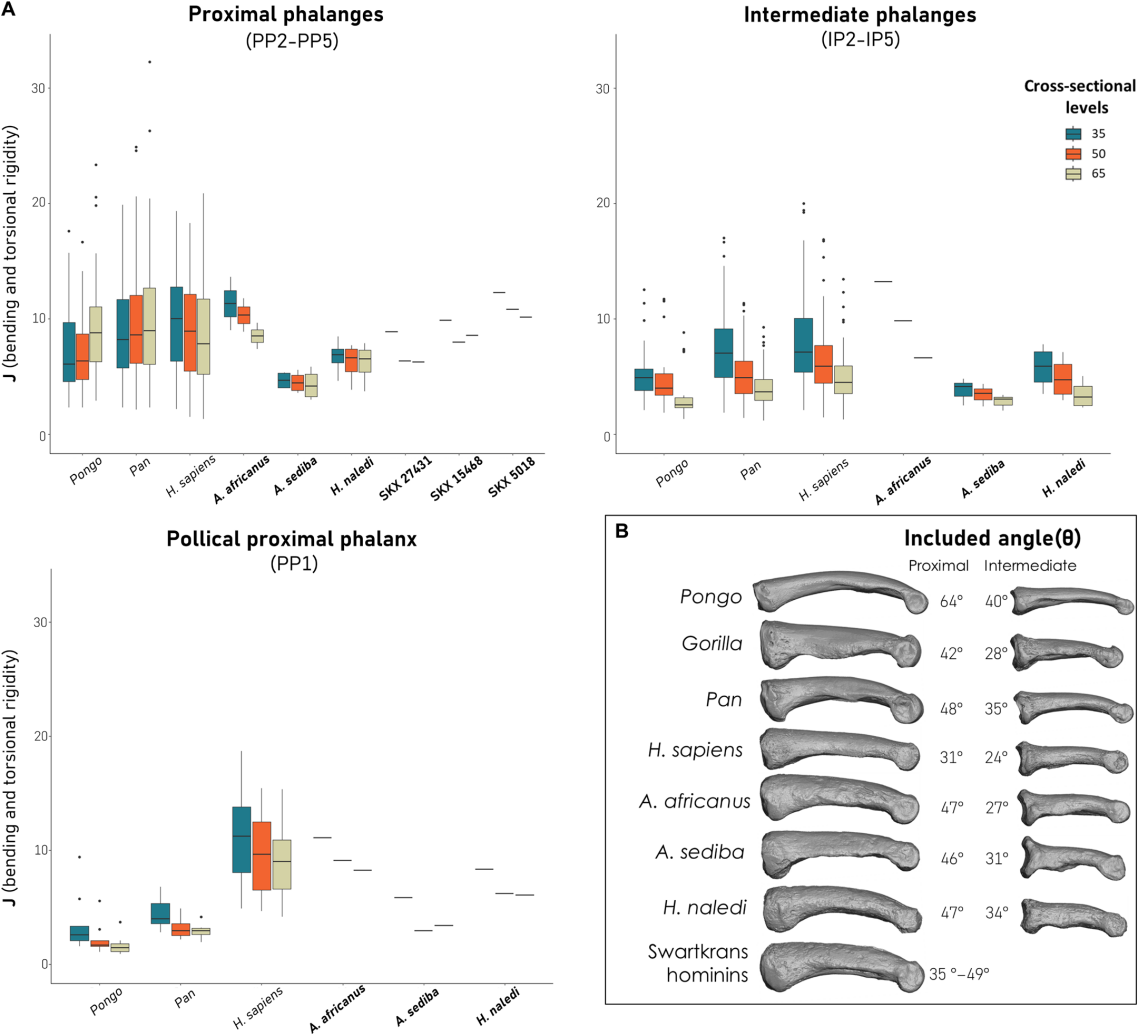
Standardized average *J* and phalangeal curvature of *A. sediba* and *H. naledi* in relation to an extant and fossil comparative sample. (**A**) Average maximum bending and torsional rigidity was standardized using maximum phalangeal length. (**B**) Phalangeal curvature measured via included angle, with images depicting proximodistal curvature of the dorsal shaft in a representative third proximal and intermediate phalanx for each taxon. Reported curvature values represent the average of digits 2 to 5 for the proximal and intermediate phalanges.

We calculated *J* as a measure of bending and torsional rigidity at cross sections taken at 35, 50, and 65% of phalangeal length in the phalanges across our sample and standardized the data by maximum proximodistal phalangeal length. *Gorilla* have significantly higher torsional strength than the remainder of our sample (fig. S6) (*48*–*50*), and thus, we have removed them to better visualize the variation across the other taxa (Fig. 4). Modern humans, *Pan* and *Pongo* show similar standardized bending and torsional rigidity values along the proximal and intermediate phalanges; however, the pattern at the proximal portion of the phalanx shaft varies. *Pan* and *Pongo* have the lowest torsional rigidity proximally (35%) in the proximal phalanx, while humans have highest values proximally and rigidity decreases distally (35% > 50% > 65% cross sections). The latter pattern is common to all extant and fossil taxa in the intermediate phalanges.

*A. sediba* has the lowest bending and torsional rigidity for both the proximal and intermediate phalanges in our fossil sample and overlaps only with the lower range of variation of our extant taxa. *H. naledi* has greater bending and torsional rigidity than *A. sediba* and falls within the interquartile range of humans and other apes. In comparison, the proximal and intermediate phalanges of *A. africanus* and, less so, the proximal phalanges from Swartkrans (at least the 35% cross section) have among the highest bending and torsional rigidity in our sample (apart from *Gorilla*; fig. S6).

Both *A. sediba* and *H. naledi* show minimal change in bending and torsional rigidity along the proximal phalanx shaft, with all three cross-sections showing similar median values, which is a pattern more similar to humans than to other apes. In the intermediate phalanges, change in bending and torsional rigidity is more notable, especially in *H. naledi*, as well as the single *A. africanus* specimen, which is more similar to the ape pattern.

### Cortical patterns in the thumb

We applied the same analyses to the pollical (thumb) proximal phalanx (PP1), and generally, the differences across extant taxa are similar to those of the fingers. All extant great apes show thick cortical bone on the distal palmar surface of the PP1 with relatively thicker cortical bone overall along the palmar shaft compared to the dorsal shaft. Modern humans also show thick cortical bone at the distal palmar surface, particularly at the attachment site of the second annular (A2) pulley that is important for the function of the flexor pollicis longus muscle (*54*), but the cortical bone is much thicker along the dorsal shaft compared to the palmar side (Fig. 1). Great apes show high scaled mean cortical thickness relative to humans that increases proximodistally along the shaft, while in humans (and *Pongo*), cortical thickness decreases after ~75% of shaft length (Fig. 3).

The *A. sediba* PP1 shows an ape-like pattern, with thick distopalmar cortical bone and relatively thin dorsal shaft cortical bone (Fig. 1) and, in the PCA, falls within and closest to the *Gorilla* distribution (Fig. 2). The typicality probability values support this result, with *A. sediba* having the closest affiliation to *Gorilla* (table S3). Its scaled mean cortical thickness is most similar to *Pongo* and the change in thickness across the shaft follows the pattern found in *Pongo* and humans (Fig. 3). The *H. naledi* PP1 cortical bone distribution pattern most closely resembles the modern human pattern due to the relatively thicker cortex on the dorsal shaft. It also has human-like, thick cortex distopalmarly, although this is found only on the radial side. Within the PCA, the *H. naledi* PP1 falls just outside the modern human distribution and demonstrates the highest typicality probability with humans (Fig. 3 and table S3). Its average scaled cortical thickness is thinner than *A. sediba* and most similar to *Pongo* but falls within the ranges of variation of all extant taxa. Change in the thickness across the *H. naledi* PP1 shaft peaks at midshaft, as in humans, but does not decrease distally as found in humans, *Pongo*, and *A. sediba*. In comparison, the *A. africanus* PP1 has the highest mean cortical thickness among our fossil sample and shows an African ape-like pattern of increasing thickness proximodistally along the shaft (Fig. 3).

Standardized bending and torsional rigidity of the extant PP1s show that *Pongo* has the lowest values, while modern humans have the highest, excluding *Gorilla* (fig. S6), and *Pan* is intermediate between the two. Bending and torsional rigidity in the extant taxa is highest at the proximal cross section (35%) and decreases distally. *A. sediba* has the lowest bending and torsional rigidity in our fossil sample and falls in the *Pan* range and lower range of modern humans. *H. naledi* has greater bending and torsional rigidity than *A. sediba* and falls within the range of modern humans. Relative to *A. sediba* and *H. naledi*, *A. africanus* has the highest bending and torsional rigidity and falls within the upper range of modern human variation (Fig. 4).

## DISCUSSION

Variation in the cortical bone structure of the proximal and intermediate phalanges among extant hominids distinguishes modern humans, with hands used primarily for manipulation, from those of great apes, in which the hand habitually incurs high loading from locomotion (*48*–*50*). Great apes show thicker cortical bone along the palmar shaft, particularly at the flexor sheath ridges of the fingers, as well as relatively thick cortical bone that increases distally along the shaft. This morphology is consistent with high loading from finger flexor tendons, generated during flexed-finger grasping (*55*), likely during arboreal locomotion as this pattern is common to both the proximal and intermediate finger phalanges of *Gorilla*, *Pan* and, to a lesser extent, *Pongo* (Fig. 3). In contrast, humans show a distinct pattern of thicker cortical bone along the dorsal shaft but relatively thin cortical bone overall that we interpret as reflecting comparatively low loading of relatively straight phalanges during manipulation (*48*–*50*). The human proximal phalanx of the thumb also has thicker dorsal shaft cortical bone and, relative to apes, thicker cortical bone at the attachment site of the A2 pulley for the flexor pollicis longus muscle tendon, a muscle that is consistently well developed in humans to the exclusion of other apes [but see (*56*)] and is active during tool-use activities (*57*, *58*). The association between differences in phalangeal cortical bone structure and differences in hand use across extant hominids supports using cortical bone as a plastic ecophenotypic trait to infer behavior in the fossil record (*48*–*50*).

Within this comparative context, *A. sediba* and *H. naledi* differ from each other, as well as from the isolated South African hominin phalanges, in their patterns of cortical bone thickness distribution and cross-sectional properties. These patterns indicate variation in phalangeal loading that, in turn, suggest differences in manual behaviors across these fossil taxa, including the functional importance of their ape-like phalangeal morphological features. We propose that this variation reflects multiple behavioral solutions to manipulative and locomotor hand use between approximately 2.0 and 0.3 Ma rather than a linear trajectory toward increasing dexterity.

### Australopithecus sediba

Cortical bone morphology of the *A. sediba* MH2 proximal and intermediate phalanges largely resembles that of great apes, signaling a hand used primarily for locomotion, but with slight variations across the digits. The African ape-like curvature (Fig. 4B) and prominent flexor sheath ridges on both the proximal and, especially, intermediate phalanges, have been interpreted as functional adaptations for flexed-finger grasping during locomotion (*6*, *34*). Here, we show that these external features were likely functionally relevant as the thick palmar cortical bone distribution and thick African ape-like mean cortical thickness reveal that the MH2 fingers and pollical phalanx were most likely loaded in locomotion during life. These functional signals from the MH2 phalanges are consistent with the trabecular bone distribution of the capitate, which suggests human-like loading combined with *Pan*-like extended wrist postures (*13*), and with the trabeculae of the ulnar metacarpal (Mc2-Mc5) heads, which is consistent with habitual flexed-finger, arboreal grasping similar to that of *Pongo* (*15*). Although the MH2 proximal phalanges have low relative bending and torsional rigidity, *Pongo* also does despite being the most arboreal of the great apes (Fig. 4). In *Pongo*, low bending and torsional rigidity likely reflects the strong longitudinal curvature of their phalanges and well-developed flexor sheath ridges that are shown to biomechanically reduce strain experienced by the bone (*30*). Similar biomechanical mechanisms would apply to the *Pan*-like curvature and well-developed flexor sheath ridges of *A. sediba* phalanges. Locomotor loading of the fingers is also supported by the morphology of the associated, complete upper limb of MH2, such as superiorly oriented glenoid fossa on the scapula, African ape-like clavicle length and orientation, relatively long arms, a relatively long ulna with keeling of the trochlear notch, well-developed wrist and finger flexor muscle entheses, and robust non-pollical metacarpal shafts (*15*, *38*, *59*, *60*).

In contrast to the morphological signals of locomotor loading in the *A. sediba* hand, the human-like distodorsal thickening of the fifth digit phalanges may reflect hand use during manipulation. Similarly, previous studies of the trabecular structure of the pollical metacarpal, which contrasts with the internal cortical structure of the pollical phalanx, suggest that it was habitually used in human-like abducted and opposed postures characteristic of forceful precision grips (*1*, *6*, *15*, *18*, *34*, *61*). These internal features can be also considered within the context of the remarkably long, albeit gracile, thumb relative to the fingers in the MH2 hand, which would have facilitated pad-to-pad precision grip (*62*) and its notably robust fifth metacarpal base and head (*6*, *34*).

African ape locomotor experimental studies show that the fifth digit experiences the lowest load during knuckle-walking while the thumb is not loaded at all (*63*, *64*). Similarly, during arboreal climbing and suspension peak pressure is at the midline of the hand (e.g., digit 3), whereas digit 5 and the thumb incur much smaller loads (*65*). Moreover, experimental studies of human Lower Paleolithic stone tool making show that the fifth digit is used and loaded at frequencies greater or equal to the thumb (*66*). Thus, the first and fifth digits may be more likely to reflect signals of manipulation in a hominin still using its hands for locomotion if forceful manipulation was routinely used in its behavioral repertoire. However, the African ape-like cortical structure of the pollical proximal phalanx, in addition to the gracile morphology of the MH2 first metacarpal (*5*, *14*), indicates that the thumb was likely not as powerful or forcefully loaded as in *H. naledi* or some other hominins (e.g., the robust first metacarpals from Swartkrans) (*67*).

We suggest that the morphology of the *A. sediba* thumb and fifth digit are best interpreted as functional signals of manipulation (e.g., five-jaw chuck or power squeeze grips that involve both of these digits) (*1*) with more limited force than *H. naledi* in a hand that was still habitually used for locomotion. Despite the human-like manipulation signal in the fifth digit, the grips used by *A. sediba* likely differed from extant analogs due to the presence of great ape-like palmar cortical thickness. That being said, the *A. sediba* phalangeal cortical bone pattern also differs from that of *A. africanus* and the Swartkrans phalanges. Although phalanges from these other taxa are isolated and digit attribution is not certain, their cortical structure is more similar to that of great apes, particularly in *A. africanus* SKX 5018 and SKX 15468, indicating a stronger locomotor signal than that of *A. sediba*.

### Homo naledi

The cortical bone of the *H. naledi* phalanges is distinct from that of *A. sediba* and the remainder of our comparative sample, which is consistent with differences in external morphology between these two taxa. In particular, *H. naledi* has cortical morphology that suggests that the proximal phalanges were loaded differently from the intermediate phalanges. The proximal phalanges not only have thick palmar cortical bone similar to great apes but also show thick dorsal cortical bone that is only found in humans. The *H. naledi* pollical proximal phalanx also shows human-like cortical bone structure, with a thick dorsal shaft and potentially asymmetrical loading of the A2 pulley for the flexor pollicis longus tendon, suggesting strong flexion and abduction of the distal thumb. These functional signals are also consistent with the external morphology of the thumb, including well-developed muscle flanges, and provide further support for a potentially powerful thumb capable of human-like manipulation (*5*, *14*).

In contrast to the human-like proximal phalanges, the intermediate phalanges show great ape-like thick palmar cortical bone (without dorsal shaft thickening), together with well-developed flexor sheath ridges and, remarkably, Asian ape-like curvature (*5*). Together, this morphology suggests a more frequent use of the fingers for locomotion. This is an unusual morphological pattern from a biomechanical perspective as it is expected that if the intermediate phalanges are resisting loads associated with grasping of substrates during locomotion, the proximal phalanges would have to be loaded in a similar manner. In bonobos, the intermediate phalanges are typically not loaded during arboreal locomotor grasping, and instead the proximal and, particularly, distal phalanges incur highest loads across the fingers (*65*).

Therefore, one hypothesis for this unusual pattern—human-like thick dorsal cortical bone in the proximal phalanges, more ape-like cortical bone in the intermediate phalanges, combined with ape-like curvature in both sets of phalanges—is that *H. naledi* was habitually using a hand grip that highly loaded the proximal and intermediate phalanges in different ways, such as crimp grips. Crimp grips are frequently used by modern humans during rock climbing (*68*–*70*), a behavior that has been previously proposed by Everett *et al.* (*68*) to explain high phalangeal curvature in *H. naledi*. Rock climbing in modern humans results in high forces across the flexor system of the hand, especially at the annular pulleys that hold the tendon close to the bone and provide mechanical advantage during flexion at the phalangeal joints (*54*, *70*–*72*). This loading is expected to increase loads, and thus cortical thickness, on the palmar shaft of the phalanges, creating compression palmarly and tension dorsally along the phalangeal shaft [(*30*) and fig. S7]. During full crimp grips, in particular, the proximal interphalangeal joint is flexed and the distal interphalangeal joint is hyperextended, resulting in palmar bending of the intermediate phalanges and dorsal bending of the proximal phalanges [(*30*) and fig. S7]. As bone is more resistant to compressive loads, the unusually high tensile loads on the dorsal shaft of the proximal phalanges may stimulate thicker dorsal cortical bone, as found in *H. naledi*. Whether crimp-like grips were used habitually by *H. naledi* and whether they were used to climb vertical rock surfaces, however, require further testing. Among modern humans, frequent recreational rock climbers have significantly higher cortical area and bending strength of their phalanges (and metacarpals) compared to non-climbers (*73*), but whether it results in thicker cortical bone on the palmar shaft specifically has not been investigated. Furthermore, several primate species frequently rock climb, such as chacma baboons [*Papio ursinus*; (*69*)] or white-headed langurs [*Trachypithecus leucocephalus*; (*74*)], but potential functional adaptations to their phalangeal morphology has not been explored. Detailed experimental and morphological investigations are needed to test if crimp-like grips and rock climbing were a part of the behavioral repertoire of *H. naledi*, but regardless of the type of locomotor behavior, it is clear that the locomotor grasping of *H. naledi* was distinct from that of *A. sediba* and likely other South African hominins.

Our results on phalangeal cortical bone morphology provide evidence for a diversity of manual behaviors in South African hominins in the Plio-Pleistocene and indicate that the primitive, great ape-like features in the hands of these hominins were functionally important rather than nonfunctional, phylogenetic retentions. The different internal (and external) phalangeal morphology of *A. sediba* MH2 hand and the *H. naledi* hand 1 represents, at least in part, differences in finger and thumb loading during life that suggest divergent solutions to the problem of balancing locomotory and manipulatory demands on the hands. These divergent solutions are perhaps expected, given that these two hominins are now separated by approximately 1.5 million years, with *A. sediba* being contemporaneous with Oldowan tool technologies (*75*) and *H. naledi* living at the beginning of the Middle Stone Age (*76*) (noting, however, that neither hominin species has yet been directly associated with stone tools). This diversity is also evident in the combinations of external morphological features that characterize *A. sediba* and *H. naledi*, but the plastic “ecophenotypic” features revealed in this study build upon previous work on these taxa [e.g., (*13*, *15*)] and provide a more refined understanding of how these individuals were interacting with their respective palaeoenvironments during life. Future experimental and computational analyses of load and strain incurred during different manipulative and locomotor activities will shed further light on the potential biomechanical demands placed on the digits and hominin hand overall and the diversity of morphology that we find in the hominin fossil record.

## METHODS

### Materials

The fossil sample included phalangeal specimens attributed to an *A. sediba* individual and an *H. naledi* individual. *A. sediba* is represented by all eight non-pollical proximal and intermediate phalanges, except the second intermediate phalanx, of the right MH2 hand (*6*, *34*). The *H. naledi* sample consists of all eight non-pollical proximal and intermediate phalanges of hand 1 (*5*). The *A. africanus* sample consisted of two isolated proximal phalanges (StW 293 and StW 122) and an intermediate phalanx (StW 331), likely belonging to different individuals. The sample from Swartkrans consisted of three isolated proximal phalanges (SKX 27431, SKX 5018, and SKX 15468) attributed to either *P. robustus/*early *Homo*. The lack of associated remains of *A. africanus* and the Swartkrans specimens make it challenging to accurately assign digit number to the individual phalanges. All of the fossil specimens used in this study are curated at the Evolutionary Studies Institute, University of the Witwatersrand, South Africa.

The extant comparative sample used in this study consists of proximal and intermediate phalanges from 92 great ape and human individuals (*Pongo* = 9, *Gorilla* = 25, *Pan* = 24, and *Homo* = 33). Great ape specimens are curated at the Royal Museum for Central Africa, Max Planck Institute for Evolutionary Anthropology, Powell-Cotton Museum, Berlin Natural History Museum, Senckenberg Natural History Museum, and the Bavarian State Collection of Zoology. The modern *Homo sapiens* specimens represent post-industrial and nonindustrial groups and are curated at the Vienna Natural History Museum, the Sackler School of Medicine at Tel Aviv University, University of Florence, the Anthropology Collection at the Georg-August-University Goettingen, the Museo Nazionale Preistorico dei Balzi Rossi, the Museo Archeologico del Finale, the Duckworth Collection at the University of Cambridge, the Mary Rose Trust, and the Czech Republic Institute of Archaeology. This extant sample studied here is a combination of the datasets reported previously (*48*–*50*), and details are reported in table S1.

### micro-CT and image segmentation

This study used high-resolution micro-computed tomography (micro-CT) scans of extant and fossil specimens. The specimens were scanned using a BIR ACTIS 225/300, Diondo D3 or Skyscan 1172 scanner housed at the Department of Human Evolution, Max Planck Institute for Evolutionary Anthropology, Germany; a Nikon 225/XTH scanner at the Cambridge Biotomography Centre, University of Cambridge, United Kingdom; or with the Diondo D1 scanner at the Imaging Centre for Life Sciences University of Kent, United Kingdom. The scans were conducted at voltages ranging from 100 to 160 kV and 100 to 140 μA, alongside the use of 0.2- to 0.5-mm copper or brass filter. The resolution of the resulting scans ranged between 0.018 to 0.044 mm, depending on the size of the bone. All scans were processed to remove soft tissue or other non-bone materials and were reoriented into standard anatomical position using Avizo Lite 9.0.0 (Visualization Sciences Group, SAS). Medical image analysis (MIA) (*77*) was subsequently used on the clean micro-CT scans to segment the bone.

Segmentation of the micro-CT scans relies on the grayscale values corresponding to the materials within the scans. This process is a challenging task when multiple materials with varying densities and voxel values are present, as was the case for the fossil specimens analyzed here. The preservation of each fossil specimen varied due to differing taphonomic conditions. Consequently, unlike the extant sample, which was segmented solely using the MIA method, the fossil specimens underwent an initial segmentation in MIA, followed by the application of image filters and manual cleaning within Avizo 6.3 (Visualization Sciences Group, SAS).

### Cortical bone analysis

The cortical bone analysis methods used in this study are consistent with those described in Syeda (*48*) and Syeda *et al.* (*49*, *50*); therefore, we provide a brief overview of the methods below. Following the segmentation of the micro-CT data, three-dimensional (3D) external and internal surfaces of the phalangeal specimens were creating using Medtool v 4.5 (www.dr-pahr.at/html/01.html) (*78*, *79*). Within Medtool, 3D volumetric masks of the outer and inner area of the cortex were created through a combination of 3D casting of rays and use of morphological filters (*78*, *79*), which are then used to create smooth external and internal surfaces within Paraview v4.4 and Meshlab v2020.03. Since our study focuses on the cortical bone of the phalangeal shaft, we extracted the defined phalangeal shaft (*48*–*50*) from the whole-bone external and internal surfaces. These defined surfaces were then input into the R package Morphomap (*53*) for cortical bone analysis. In Morphomap, 97 cross sections, spaced at 1% intervals, were extracted from 2 to 98% of the phalangeal shaft length. On each cross section, 50 equiangular semi-landmarks were to capture the shape of the shaft. The number of cross sections and semi-landmarks used in this study are consistent with previously published data to ensure comparability between the fossil and extant sample. For each cross section, rays were projected outward from the centroid to each pair of equiangular semi-landmarks (located on the outer and inner surfaces), and cortical thickness was calculated as the length between the landmarks on the external and internal surface. The cortical bone thickness data were then used to generate morphometric maps representing the cortical bone distribution patterns of each individual phalanx. As Morphomap also calculates cross-sectional geometric properties, we quantified the polar second moment of area, which is a measure of bending and torsional rigidity, at 35, 50, and 65% of the phalangeal length. Because of differences in overall phalangeal size across our sample, we standardized the cortical thickness values and bending and torsional rigidity values by the maximum length of each phalanx. Maximum phalangeal length was measured in Avizo Lite 9.0.0 (Visualization Sciences Group, SAS) using the 3D external surfaces of each specimen, taking maximum length from the distal most extent of the trochlea to the proximal most extent of the base in dorsal view.

### Statistical analysis

To analyze cortical bone distribution of the phalangeal shaft, PCAs were performed on the cortical thickness values of the phalangeal shaft. As analyses on the extant sample have been conducted [see (*50*, *51*) for details], we only conducted tests on fossil specimens to determine whether they were significantly different from the extant comparative sample. Hotelling one sample T-squared tests were conducted on the fossil sampled to provide statistical power to the results visualized in the PCA. Further testing was conducted on the PC scores to assess the relationship between the fossil specimens and extant groups. Canonical variates analyses (CVAs) were conducted on the first three PC scores of each PCA to visualize the accuracy of extant group separation. The results of the CVA were further used to calculate the typicality probability for each fossil specimen, offering statistical insights into the extent to which each fossil aligns with a particular extant group. The PCA was performed using the “prcomp” function in the R Stats package, the hotelling one-sample T-squared tests were conducted using R package ICSNP, and the CVA and typicality probability were calculated using R package Morpho.
