# Supplementary material for: Phalangeal cortical bone distribution reveals different dexterous and climbing behaviors in Australopithecus sediba and Homo naledi
Source: Sci Adv. 2025 May 14;11(20):eadt1201. doi: 10.1126/sciadv.adt1201 (PMC12077519; doi:10.1126/sciadv.adt1201)
Supplement: Supplementary file 1 — Figs. S1 to S7 Tables S1 to S3 [file sciadv.adt1201_sm.pdf]

Science Advances
AAAS

Supplementary Materials for

Phalangeal cortical bone distribution reveals different dexterous and climbing behaviors in *Australopithecus sediba* and *Homo naledi*

Samar M. Syeda *et al.*

Corresponding author: Samar M. Syeda, ssyeda@amnh.org

Sci. Adv. **11**, eadt1201 (2025)
DOI: 10.1126/sciadv.adt1201

This PDF file includes:

Figs. S1 to S7
Tables S1 to S3

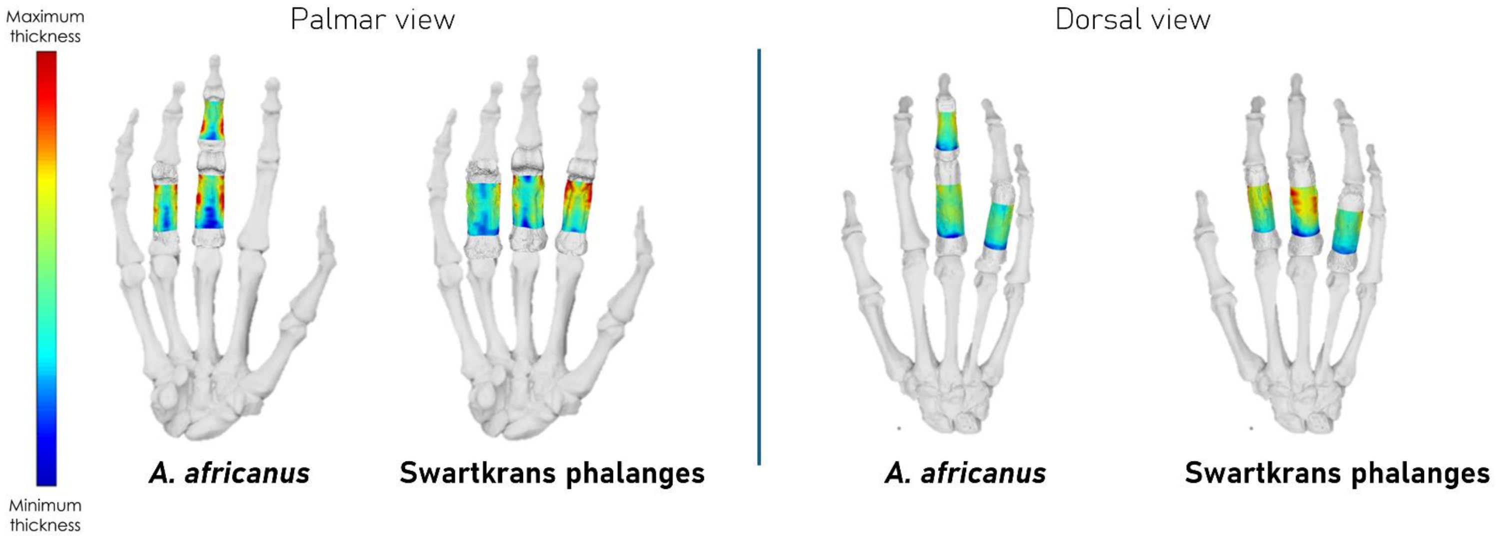

Supplementary Figures

Fig. S1.

3D colour maps of cortical bone distribution in *A. africanus* and in the Swartkrans phalanges. The thickness maps are depicted on the same hand model for visualisation purposes only. The phalanges are isolated, with no digit number confidently attributed to them. *A. africanus* phalanges: IP = StW 331, PPs from left to right in palmar view = StW 293, StW 122; Swartkrans phalanges, from left to right in palmar view: SKX 27431, SKX 5018, SKX 15468.

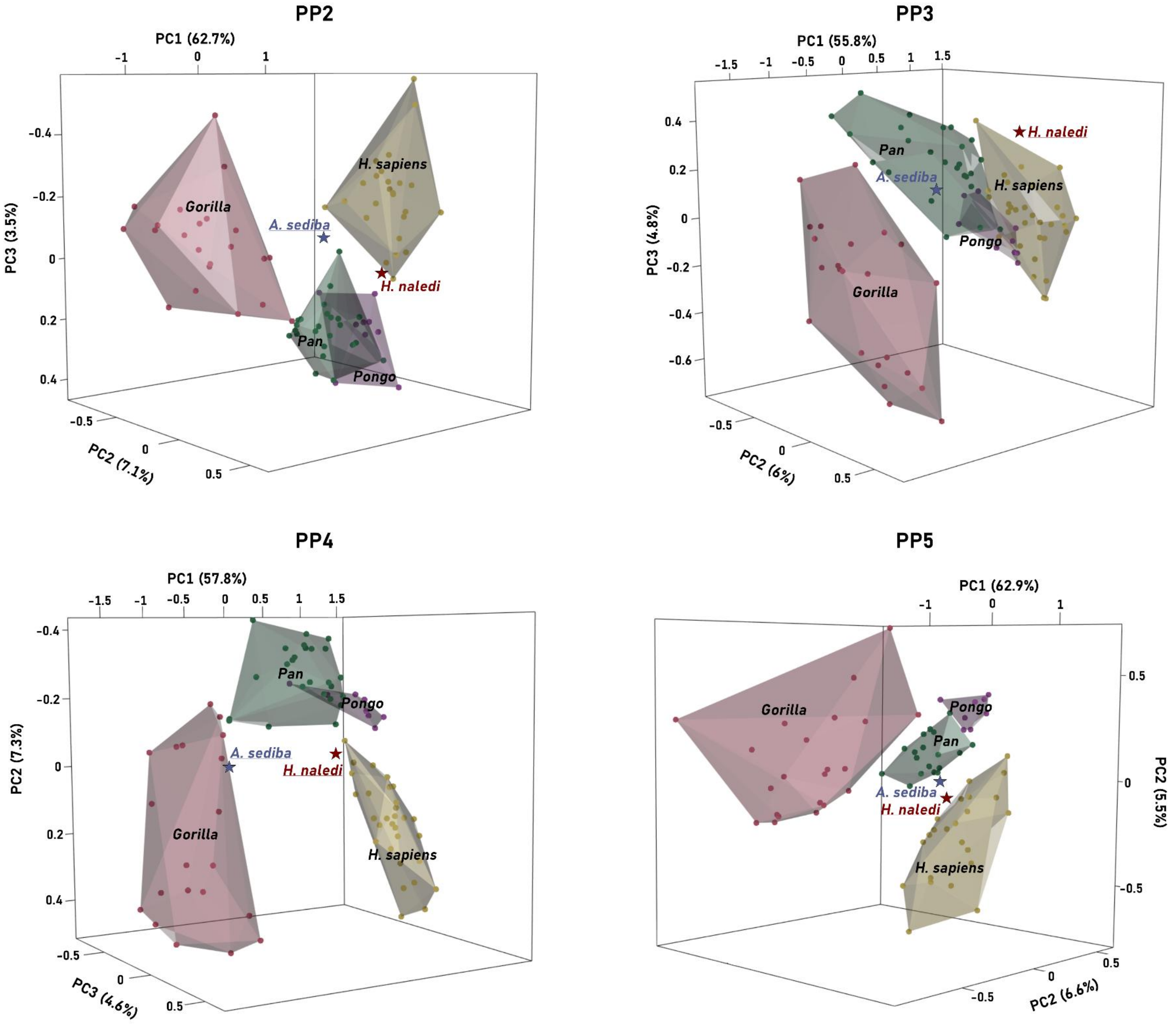

Fig. S2.

Variation in cortical bone distribution of each proximal phalanx in multivariate shape space in fossil and extant hominids. 3D PCAs depicting variation in cortical bone distribution across the proximal phalanges.

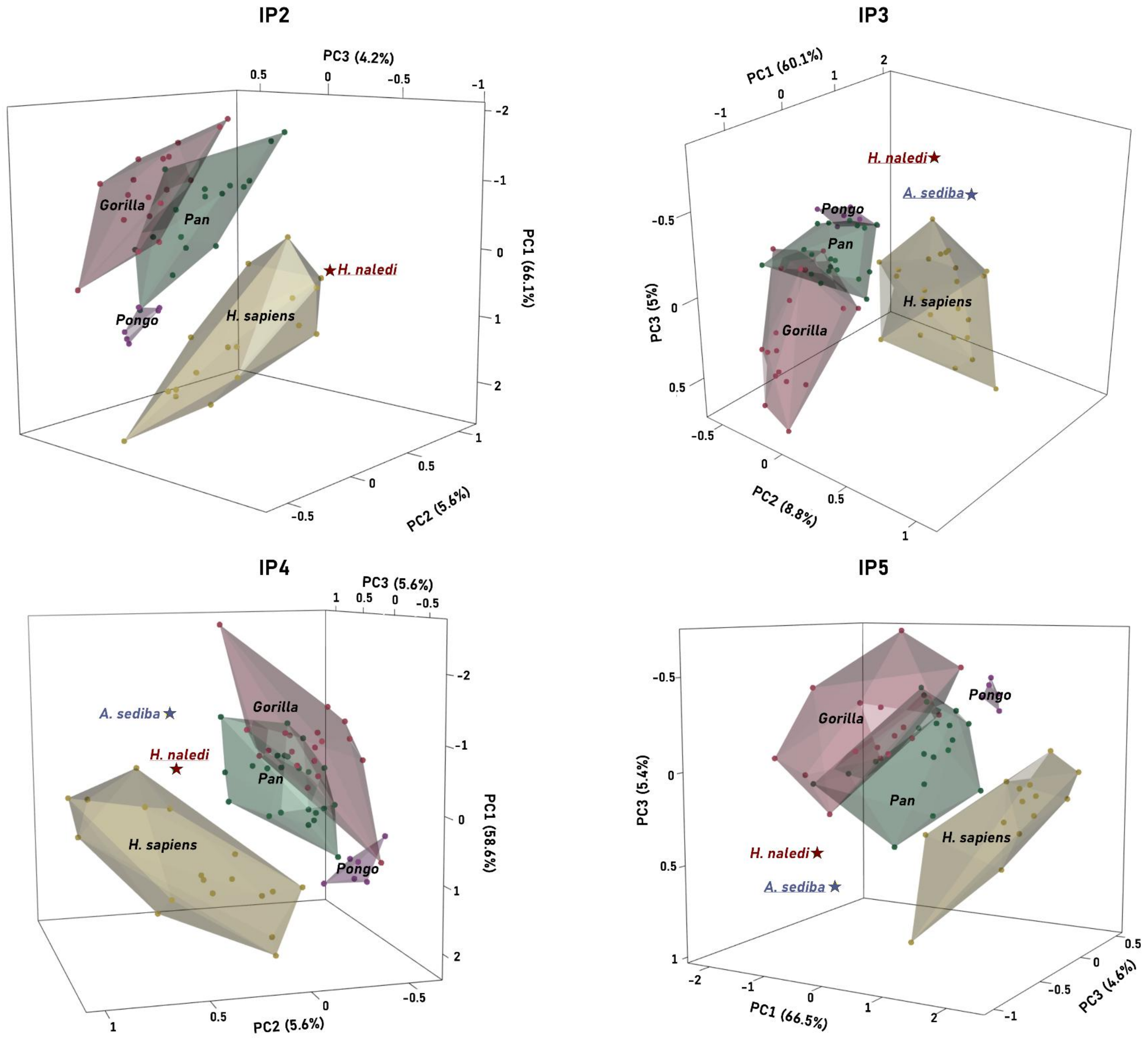

Fig. S3.

Variation in cortical bone distribution of each intermediate phalanx in multivariate shape space in fossil and extant hominids. 3D PCAs depicting variation in cortical bone distribution across the intermediate phalanges.

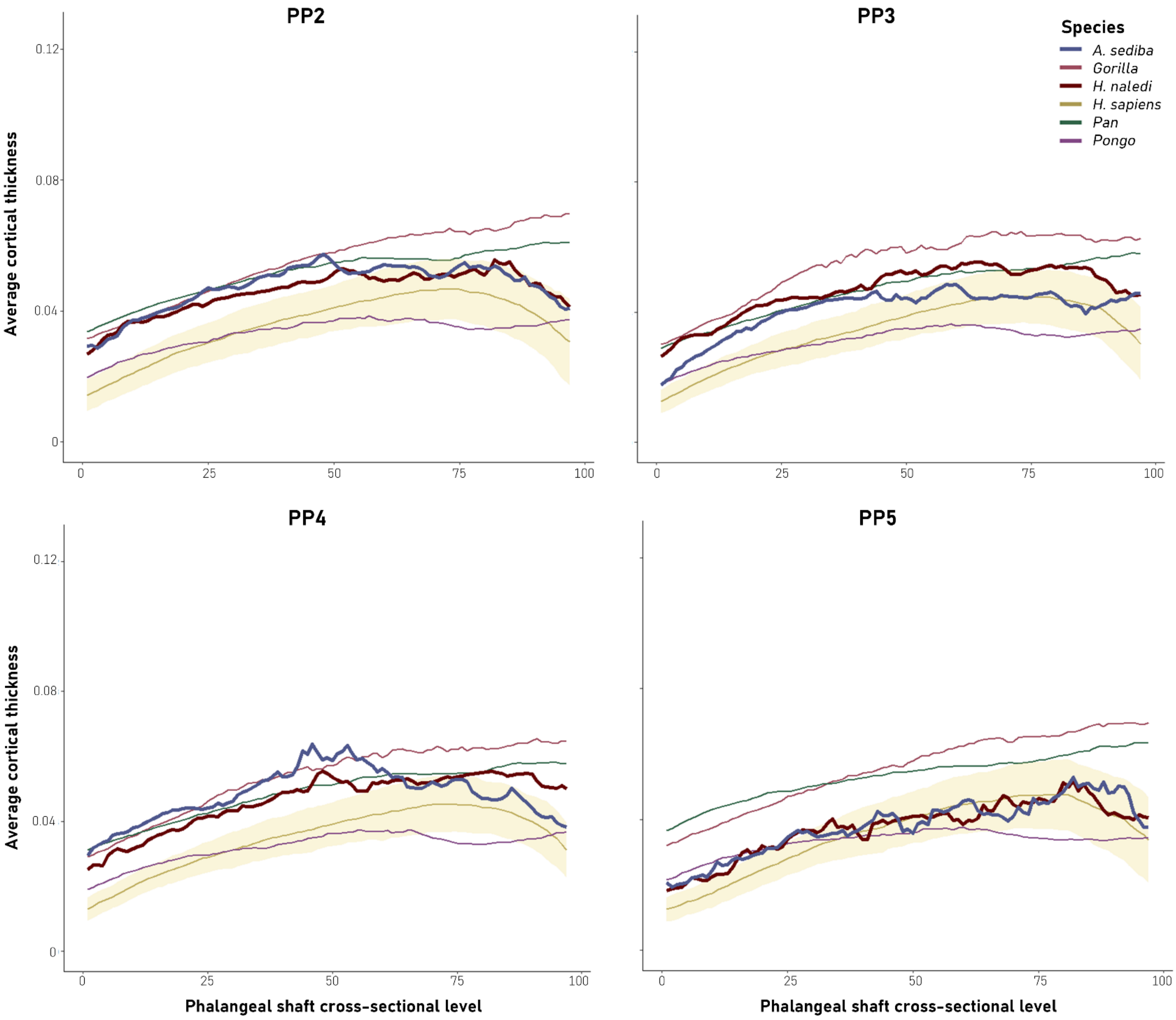

Fig. S4.

Average cortical bone thickness across the phalangeal shaft. Cortical bone thickness is plotted from the proximal end (0) to the distal end (100) of the defined phalangeal shaft for each proximal phalanx. The shaded region around the *H. sapiens* average represents the variation in the *H. sapiens* sample. Fossil taxa are depicted with bolder lines.

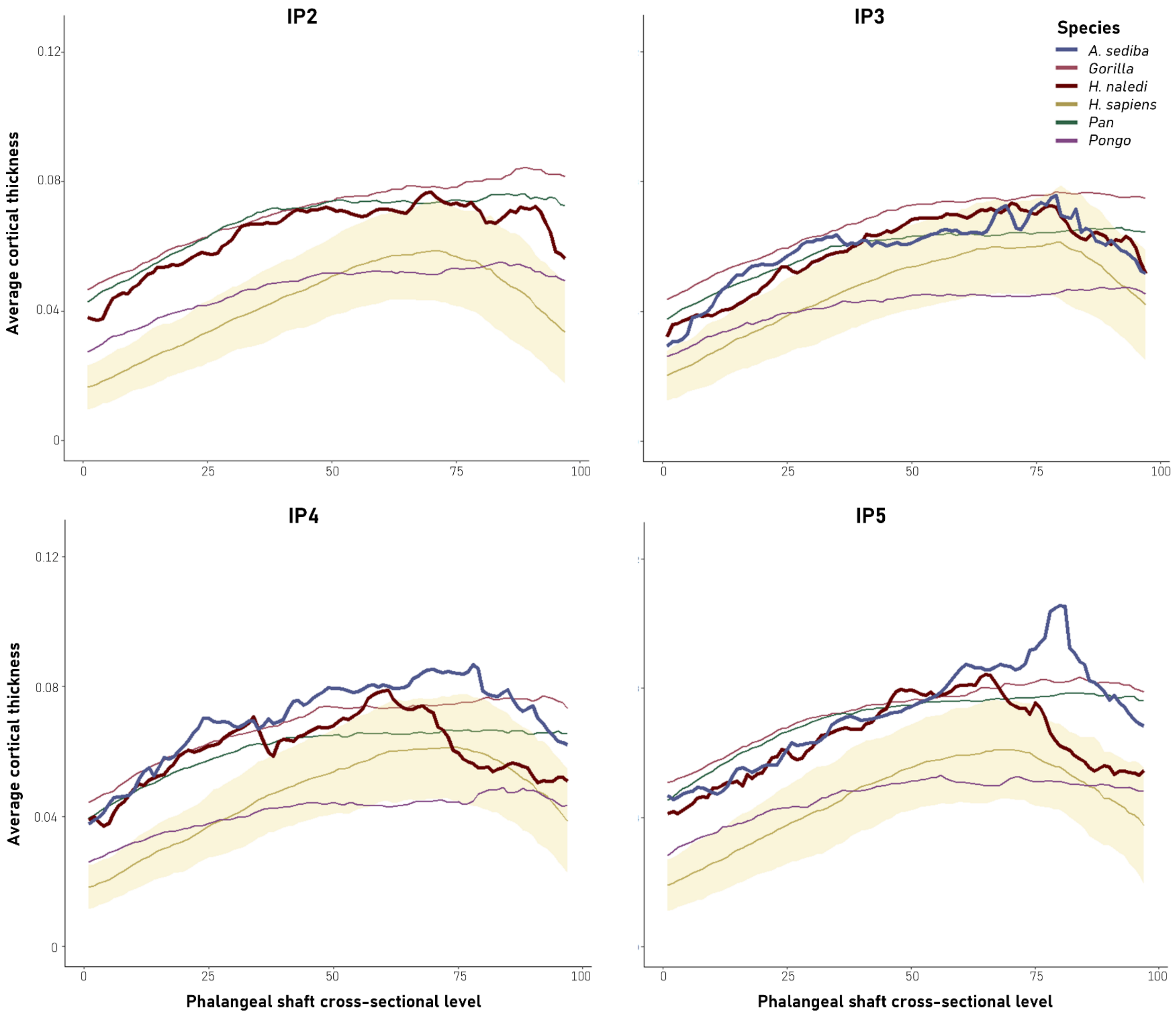

Fig. S5.

Average cortical bone thickness across the phalangeal shaft. Cortical bone thickness is plotted from the proximal end (0) to the distal end (100) of the defined phalangeal shaft for each intermediate phalanx. The shaded region around the *H. sapiens* average represents the variation in the *H. sapiens* sample. Fossil taxa are depicted with bolder lines.

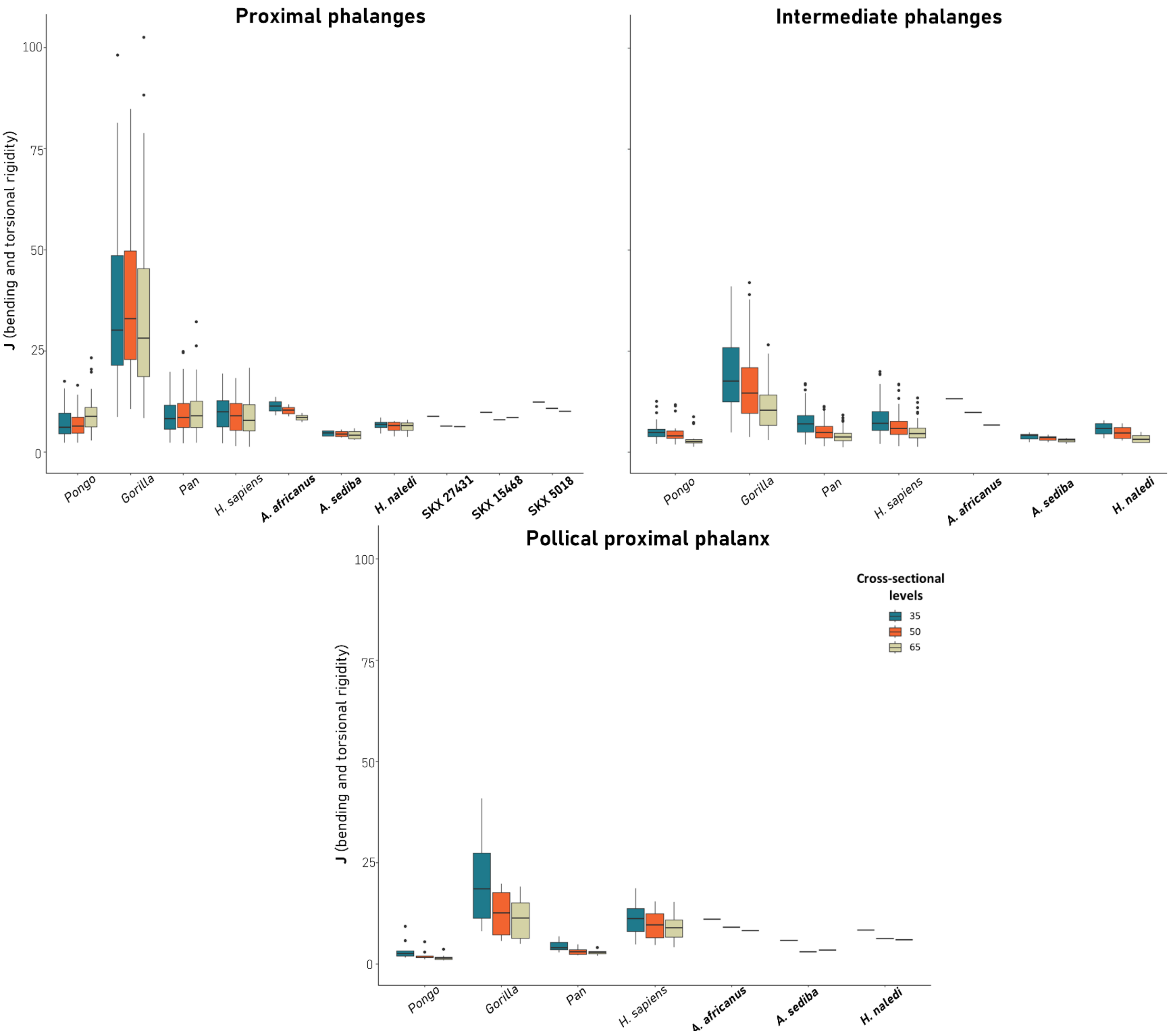

Fig. S6.

Standardised average maximum bending and torsional rigidity. Average maximum bending and torsional rigidity plots with *Gorilla* included.

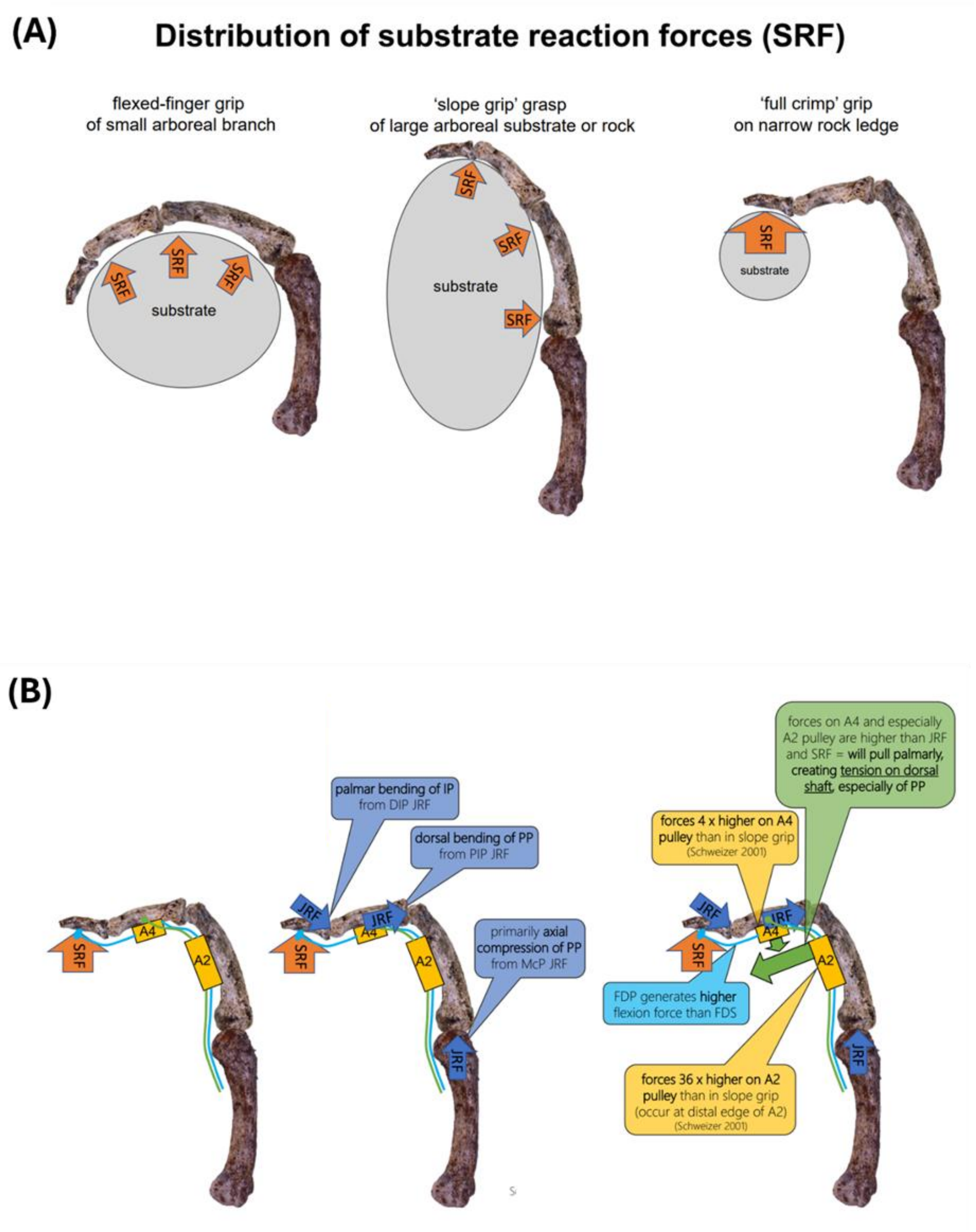

Fig. S7.

Schematic demonstrating the loads experienced by the fingers during various rock-climbing grips. (A) Substrate reaction forces (SRF) experienced by the phalanges during flexed finger grips, slope grip, and full crimp grip. **(B)** Biomechanical loads experienced by the phalanges during the use of the full crimp grip. When using this grip, loads incurred by the soft tissues (A2 and A4 pulleys) are significantly higher than the SRF and JRF (73). We hypothesize that the high pulley loads which would act to

palmarly bend the intermediate and proximal phalanges, creating tension on the dorsal shaft that stimulates bone remodeling.

Table S1.

Detailed information on extant sample.

Taxon	Specimen	Sex	Side	Subsistence	Curatorial Institution
Homo sapiens	NHMW-Nubian_J2	M	R	Pre-industrial	Natural History Museum, Vienna
Homo sapiens	NHMW-Nubian_K63	M	L	Pre-industrial	Natural History Museum, Vienna
Homo sapiens	NHMW-Nubian_K5.2	M	R	Pre-industrial	Natural History Museum, Vienna
Homo sapiens	NHMW-Nubian_J7	F	R	Pre-industrial	Natural History Museum, Vienna
Homo sapiens	Qafzeh_9	M	R	Pre-industrial	Sackler School of Medicine, Tel Aviv
Homo sapiens	Qafzeh_8	UK	R/L	Pre-industrial	Sackler School of Medicine, Tel Aviv
Homo sapiens	OHALO_II_H2	M	R/L	Pre-industrial	Sackler School of Medicine, Tel Aviv
Homo sapiens	UNIFL_4865	M	R	Post-industrial	University of Florence
Homo sapiens	UNIFL_4887	F	R	Post-industrial	University of Florence
Homo sapiens	UNIFL_3124	UK	UK	Pre-industrial	University of Florence
Homo sapiens	UNIFL_3125	UK	UK	Pre-industrial	University of Florence
Homo sapiens	UNIFL_3127	M	L	Pre-industrial	University of Florence
Homo sapiens	GAUG-Inden_91	M	R	Post-industrial	Georg-August-University Goettingen
Homo sapiens	GAUG-Inden_113	M	L	Post-industrial	Georg-August-University Goettingen
Homo sapiens	GAUG-Inden_117	UK	L	Post-industrial	Georg-August-University Goettingen
Homo sapiens	GAUG-Inden_119	M	R	Post-industrial	Georg-August-University Goettingen
Homo sapiens	GAUG-Inden_243	M	L	Post-industrial	Georg-August-University Goettingen
Homo sapiens	Barma_Grande_2	M	R	Pre-industrial	Museo Nazionale Preistorico dei Balzi Rossi
Homo sapiens	ARENE_CANDIDE_2	M	R/L	Pre-industrial	Museo Archeologico del Finale
Homo sapiens	DCW_AM_3_0_2	UK	R/L	Pre-industrial	Duckworth Collection, Cambridge
Homo sapiens	DCW_OC_1_0_141	UK	R/L	Pre-industrial	Duckworth Collection, Cambridge
Homo sapiens	DCW_OC_1_0_26	UK	L	Pre-industrial	Duckworth Collection, Cambridge
Homo sapiens	FCS8	M	R/L	Post-industrial	Mary Rose Trust
Homo sapiens	81-H1035	M	R	Post-industrial	Mary Rose Trust
Homo sapiens	81-H1040	M	R	Post-industrial	Mary Rose Trust
Homo sapiens	81-H1068-DD	M	R	Post-industrial	Mary Rose Trust

30	*Homo sapiens*	FCS16	M	R/L	Post-industrial	Mary Rose Trust
31	*Homo sapiens*	FCS17	M	R/L	Post-industrial	Mary Rose Trust
32	*Homo sapiens*	81-H172-H	M	R/L	Post-industrial	Mary Rose Trust
33	*Homo sapiens*	DV-13	M	L	Pre-industrial	Institute of Archaeology, Czech Republic
34	*Homo sapiens*	DV-14	M	R	Pre-industrial	Institute of Archaeology, Czech Republic
35	*Homo sapiens*	DV-15	M	R/L	Pre-industrial	Institute of Archaeology, Czech Republic
36	*Homo sapiens*	DV-16	M	R/L	Pre-industrial	Institute of Archaeology, Czech Republic
37	*Pan paniscus*	MRAC_27698	F	L	Wild	Royal Museum for Central Africa, Tervuren
38	*Pan paniscus*	MRAC_29042	F	R	Wild	Royal Museum for Central Africa, Tervuren
39	*Pan paniscus*	MRAC_29045	F	L	Wild	Royal Museum for Central Africa, Tervuren
40	*Pan paniscus*	MRAC_29052	M	R	Wild	Royal Museum for Central Africa, Tervuren
41	*Pan paniscus*	MRAC_27696	M	L	Wild	Royal Museum for Central Africa, Tervuren
42	*Pan paniscus*	MRAC_20881	M	L	Wild	Royal Museum for Central Africa, Tervuren
43	*Pan paniscus*	MRAC_29060	F	R	Wild	Royal Museum for Central Africa, Tervuren
44	*Pan troglodytes*	MPITC_11778	F	R	Wild	Max Planck Institute for Evolutionary Anthropology
45	*Pan troglodytes*	MPITC_14996	F	L	Wild	Max Planck Institute for Evolutionary Anthropology
46	*Pan troglodytes*	MPITC_11903	M	L	Wild	Max Planck Institute for Evolutionary Anthropology
47	*Pan troglodytes*	MPITC_11789	M	L	Wild	Max Planck Institute for Evolutionary Anthropology
48	*Pan troglodytes*	MPITC_11781	M	L	Wild	Max Planck Institute for Evolutionary Anthropology
49	*Pan troglodytes*	NH_CAM1_204	F	L	Wild	Powell-Cotton Museum
50	*Pan troglodytes*	NH_CAM2_301	F	R	Wild	Powell-Cotton Museum
51	*Pan troglodytes*	NH_MER_279	F	L	Wild	Powell-Cotton Museum
52	*Pan troglodytes*	NH_MER35_86	F	L	Wild	Powell-Cotton Museum
53	*Pan troglodytes*	NH_MER35_105	F	R	Wild	Powell-Cotton Museum
54	*Pan troglodytes*	PC_ZVII_24	M	R	Wild	Powell-Cotton Museum
55	*Pan troglodytes*	NH_MER33_712	M	R	Wild	Powell-Cotton Museum
56	*Pan troglodytes*	NH_MER33_724	M	L	Wild	Powell-Cotton Museum
57	*Pan troglodytes*	NH_MER32_401	M	L	Wild	Powell-Cotton Museum
58	*Pan troglodytes*	NH_MER33_440	M	R	Wild	Powell-Cotton Museum
59	*Pan troglodytes*	NH_MER36_254	M	L	Wild	Powell-Cotton Museum

60	*Pan troglodytes*	ZSM_AP-122	M	R	Wild	Bavarian State Collection of Zoology
61	*Gorilla gorilla*	ZMB_11642	UK	R	Wild	Natural History Museum, Berlin
62	*Gorilla gorilla*	ZMB_83545	M	R	Wild	Natural History Museum, Berlin
63	*Gorilla gorilla*	PC_MER_95	F	R	Wild	Powell-Cotton Museum
64	*Gorilla gorilla*	PC_MER_135	M	L	Wild	Powell-Cotton Museum
65	*Gorilla gorilla*	PC_MER_264	M	L	Wild	Powell-Cotton Museum
66	*Gorilla gorilla*	PC_MER_300	F	R	Wild	Powell-Cotton Museum
67	*Gorilla gorilla*	PC_MER_372	M	R	Wild	Powell-Cotton Museum
68	*Gorilla gorilla*	PC_MER_962	M	R	Wild	Powell-Cotton Museum
69	*Gorilla gorilla*	PC_MER1_29	F	R	Wild	Powell-Cotton Museum
70	*Gorilla gorilla*	PC_MER_138	F	L	Wild	Powell-Cotton Museum
71	*Gorilla gorilla*	PC_MER_174	F	R	Wild	Powell-Cotton Museum
72	*Gorilla gorilla*	PC_MER_696	F	R	Wild	Powell-Cotton Museum
73	*Gorilla gorilla*	PC_MER_856	F	L	Wild	Powell-Cotton Museum
74	*Gorilla gorilla*	PC_ZII_64	M	R	Wild	Powell-Cotton Museum
75	*Gorilla gorilla*	PC_ZVI_32	M	R	Wild	Powell-Cotton Museum
76	*Gorilla gorilla*	NH_MER33_755	F	R	Wild	Powell-Cotton Museum
77	*Gorilla gorilla*	NH_MER33_461	M	R	Wild	Powell-Cotton Museum
78	*Gorilla gorilla*	NH_CAM1_106	M	R	Wild	Powell-Cotton Museum
79	*Gorilla gorilla*	NH_CAM1_105	M	R	Wild	Powell-Cotton Museum
80	*Gorilla gorilla*	NH_MER35_150	F	R	Wild	Powell-Cotton Museum
81	*Gorilla gorilla*	NH_CAM1_98	F	L	Wild	Powell-Cotton Museum
82	*Gorilla gorilla*	NH_MER35_136	F	L	Wild	Powell-Cotton Museum
83	*Gorilla gorilla*	NH_MER35_139	F	L	Wild	Powell-Cotton Museum
84	*Gorilla gorilla*	NH_FC_130	M	L	Wild	Powell-Cotton Museum
85	*Gorilla gorilla*	NH_FC_123	M	L	Wild	Powell-Cotton Museum
86	*Pongo abelii*	SMF_6785	M	R	Wild	Senckenberg Natural History Museum
87	*Pongo abelii*	SMF_6779	F	L	Wild	Senckenberg Natural History Museum
88	*Pongo pygmaeus*	ZSM_1907_0633b	F	R	Wild	Bavarian State Collection of Zoology
89	*Pongo pygmaeus*	ZSM_1907_0629b	M	R	Wild	Bavarian State Collection of Zoology

Pongo pygmaeus	ZSM_1907_0660	F	R	Wild	Bavarian State Collection of Zoology
Pongo pygmaeus	ZSM_AP-120	M	L	Wild	Bavarian State Collection of Zoology
Pongo pygmaeus	ZSM_1907_0483	F	R	Wild	Bavarian State Collection of Zoology
Pongo pygmaeus	ZSM_1909_0801	M	R	Wild	Bavarian State Collection of Zoology
Pongo pygmaeus	ZMB_87092	F	R	Wild	Bavarian State Collection of Zoology

Note: M = male; F = female; R = right; L = left; UK = unknown sex/side; R/L = to have all four digits represented, some phalanges were from the right side of the body and some from the left

Table S2.

Statistical comparisons of fossil specimens and extant sample. Results of the permutational one-sampling Hotelling's T^2 test across the pooled non-pollical proximal phalanges, intermediate phalanges and the pollical proximal phalanx. Statistically significant ($p < 0.05$) F-values marked with an asterisk. The tests were conducted on the first three principal components of cortical bone thickness values.]

Proximal phalanges (PP2-PP5)

	A. africanus	***A. sediba***	***H. naledi***	**SKX27431**	**SKX15468**	**SKX5018**
Pongo	117.89*	52.06*	37.72*	85.82*	115.47*	113.82*
Gorilla	56.63*	118.15*	76.28*	44.69*	83.07*	76.32*
Pan	94.44*	70.35*	59.07*	68.89*	91.82*	90.43*
H. sapiens	122.18*	107.09*	125.50*	140.18*	116.52*	122.76*

Intermediate phalanges (IP2-IP5)

	A. africanus	***A. sediba***	***H. naledi***
Pongo	118.31*	269.66*	318.54*
Gorilla	36.96*	44.74*	32.15*
Pan	17.72*	11.15*	11.41*
H. sapiens	98.21*	106.34*	98.76*

Pollical proximal phalanx (PP1)

	A. africanus	***A. sediba***	***H. naledi***
Pongo	1.68	51.83*	53.74*
Gorilla	51.77*	26.47*	96.08*
Pan	78.16*	67.92*	81.11*
H. sapiens	77.13*	164.24*	24.71*

Table S3.

Typicality probability of fossil specimens. The results of the Canonical Variates Analysis (CVA) conducted on the first three principal component (PC) scores were utilized to calculate the typicality probability for each fossil specimen, offering statistical evidence for the likelihood that the specimens align with a specific extant group.

Proximal phalanges (PP2-PP5)

	Pongo	*Gorilla*	*Pan*	*H. sapiens*
**Stw 122**	<0.001	<0.001	<0.001	<0.001
**Stw 293**	<0.001	<0.001	<0.001	<0.001
A. sediba	0.063	0.018	0.058	0.001
H. naledi	0.090	<0.001	0.050	0.361
**SKX 27431**	<0.001	<0.001	<0.001	<0.001
**SKX 15468**	<0.001	<0.001	<0.001	<0.001
**SKX 5018**	<0.001	<0.001	<0.001	<0.001

Intermediate phalanges (IP2-IP5)

	Pongo	*Gorilla*	*Pan*	*H. sapiens*
**Stw 331**	<0.001	<0.001	<0.001	<0.001
A. sediba	<0.001	<0.001	0.006	0.002
H. naledi	<0.001	0.001	0.015	0.142

Pollical proximal phalanx (PP1)

	Pongo	*Gorilla*	*Pan*	*H. sapiens*
**Stw 575**	0.036	0.101	0.442	0.067
A. sediba	0.089	0.306	0.087	<0.001
H. naledi	0.210	0.003	0.017	0.601
